# Beyond Direct Fibrinolysis: Novel Approaches to Thrombolysis

**DOI:** 10.3390/ph19010010

**Published:** 2025-12-20

**Authors:** Alexey M. Shibeko, Nikita S. Nikitin, Nadezhda A. Podoplelova, Valentin A. Manuvera, Vassili N. Lazarev

**Affiliations:** 1 Center for Theoretical Problems of Physicochemical Pharmacology, 119991 Moscow, Russiapodoplelovan@yandex.ru (N.A.P.); 2 Federal Research and Clinical Center of Pediatric Hematology, Oncology, and Immunology, 117198 Moscow, Russia; 3 Center for Genetic Reprogramming and Gene Therapy, Lopukhin Federal Research and Clinical Center of Physical-Chemical Medicine of Federal Medical Biological Agency, 119435 Moscow, Russialazar0@mail.ru (V.N.L.); 4 Moscow Center for Advanced Studies 20, Kulakova Str., 123592 Moscow, Russia

**Keywords:** fibrinolysis, thrombolysis, thrombolytic therapy, tissue plasminogen activator, fibrin, blood clot, ADAMTS13, von Willebrand factor, NETs, DNAse

## Abstract

Fibrinolysis is a natural component of hemostasis in which a no-longer-needed clot is gradually dissolved to restore blood flow. Under pathological thrombotic conditions, this process can be pharmacologically enhanced to promote clot removal. However, thrombolytic therapy has limited efficacy and is associated with a risk of bleeding complications, including intracranial hemorrhage. Fibrinolysis targets only the fibrin-rich part of the thrombus, whereas a substantial fraction of the clot is enriched with non-fibrin components such as extracellular DNA, von Willebrand factor, and extracellular matrix proteins, including collagen, fibronectin, and laminin. These structural regions, which may constitute half or more of the clot volume, remain resistant to classical fibrinolytic agents. To overcome these limitations, recent therapeutic strategies aim to degrade these non-fibrin elements to improve thrombolytic efficacy and reduce adverse effects. In this review, we summarize current trends in pharmacological clot dissolution, discuss novel agents in clinical use and development, and outline how targeting non-fibrin components may influence the future of thrombolytic therapy.

## 1. Introduction

Cardiovascular diseases remain a leading cause of premature mortality and disability worldwide [1]. Ischemic stroke, myocardial infarction, venous thromboembolism, and pulmonary embolism together account for millions of deaths annually and contribute substantially to long-term disability. The advent of thrombolytic therapy in the late 20th century changed the principles of the acute cardiovascular and cerebrovascular care. For the first time, pharmacological agents were not only for secondary prevention and rehabilitation but also to actively dissolve obstructive clots and restore blood flow [2]. Although thrombolysis has markedly improved neurological and functional outcomes [3], it has significant limitations, including a narrow therapeutic window (within 4.5 h after stroke onset) [4]; relatively low and variable efficacy depending on clot structure and patient-specific factors; and an increased risk of major bleeding and hemorrhagic complications [5]. In this review, we summarize current advances in thrombolytic therapy, highlight efforts to overcome the existing limitations of fibrinolytic therapy, discuss new therapeutic targets within thrombus structures, and outline an integral approach in the therapy.

### 1.1. Blood Clots: Structure and Composition

**Figure 1:**
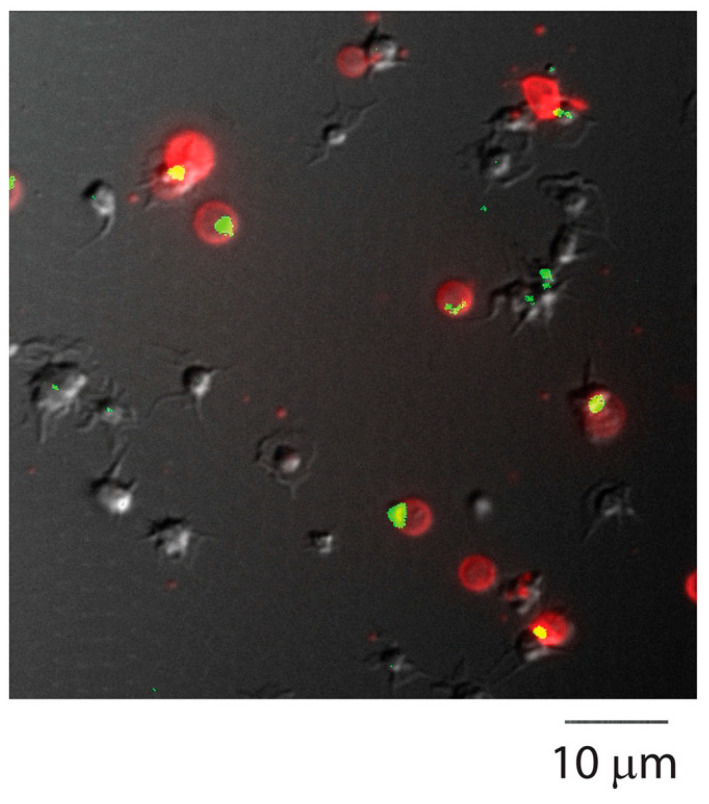
Localization of fibrin(ogen) in a single cap on the surface of the PS-positive platelets. Platelets were activated with 100 nM thrombin for 15 min and labeled with anti-fibrinogen antibody (green) and annexin V (red). Scale bar is 10 µm. Adopted from [9], CC BY 4.0.

Blood clots are complex, dynamic structures formed through the coordinated action of the blood coagulation cascade and platelet activation. Their primary physiological function is to prevent blood loss following vascular injury. However, under pathological conditions, clots may also form within intact vessels, obstructing blood flow and causing ischemic injury to the surrounding tissue. Structurally, a thrombus is not a homogeneous mass of solidified blood but a highly organized, heterogeneous composition of blood cells and proteins. Activated platelets aggregate and interact via glycoprotein receptors and adhesive ligands such as von Willebrand factor (vWF), forming initial thrombus [6]. Upon activation, some platelets expose phosphatidylserine on their surface and become so called “coated” procoagulant platelets [7,8]. These platelets accumulate fibrin(ogen) and factor XIII in the “cap” region of their surface [9,10], thereby strengthening the clot (Figure 1, which illustrates the accumulation of fibrinogen on the surface of procoagulant platelets), whereas phosphatidylserine negative platelets remain largely inert in terms of coagulation reactions.

Platelet subpopulations also differ in their adhesive and proaggregatory properties [11,12] play critical role in heterogeneous structure thrombi formation [13]. A widely accepted conceptual model describes thrombi as consisting of a densely packed, highly activated “core” surrounded by a more loosely organized, platelet-rich “shell” [14]. Fibrin, formed through the thrombin-mediated conversion of fibrinogen, provides the insoluble polymeric scaffold that stabilizes the clot [15]. Red blood cells are frequently entrapped in this network, particularly in venous thrombi, contributing to clot bulk and mechanical stability [16]. During clot contraction, erythrocytes become densely packed into polyhedral shapes (“polyhedrocytes”), which reduces clot permeability and renders the thrombus less susceptible to fibrinolysis [17].

Fibrin-rich clots are generally more responsive to plasminogen activator type drugs than platelet-rich clots. Platelets release antifibrinolytic proteins such as plasminogen activator inhibitor-1 (PAI-1) [18] and thrombin-activatable fibrinolysis inhibitor (TAFI) [19], which protect the clot from lysis [20,21]. Under shear stress, vWF and fibrinogen mediate platelet–platelet interactions [22]. Thrombus regions enriched in vWF but poor in fibrin can be particularly resistant to fibrinolysis because they lack the substrate required for plasmin generation.

Leukocytes, especially neutrophils, accumulate at the thrombus margins and release neutrophil extracellular traps (NETs), DNA-histone networks that further stiffen the thrombus and resist enzymatic breakdown [23]. NETs interact directly with fibrin and vWF, decreasing clot permeability and further hindering the diffusion and activity of fibrinolytic enzymes [24,25,26].

**Figure 2:**
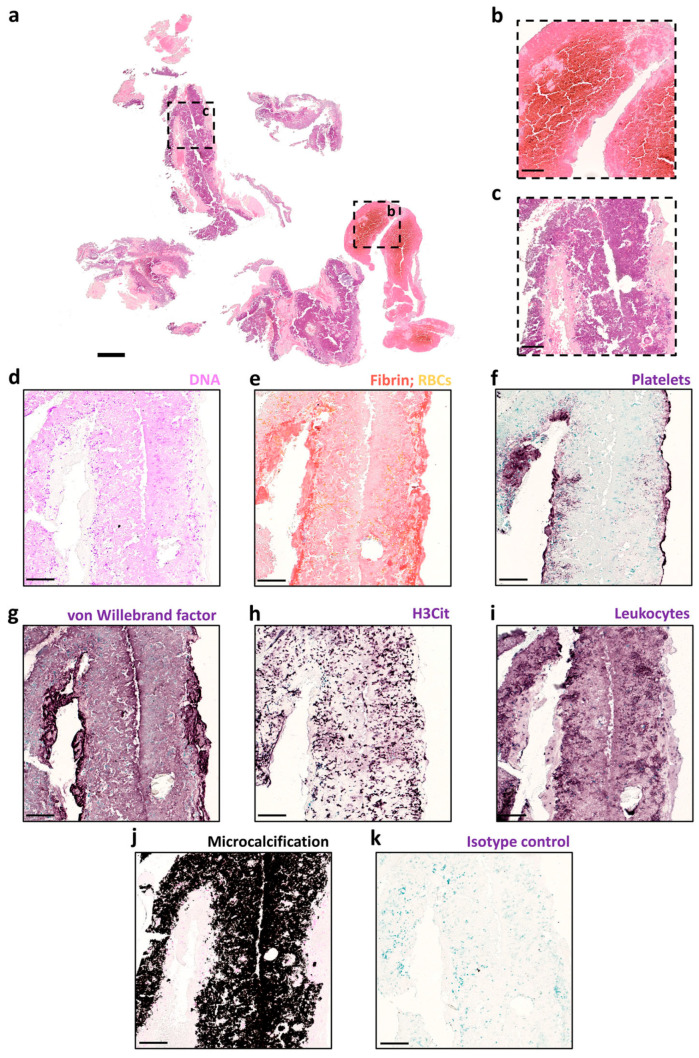
Histological analysis. The thrombus was stained for H&E, MSB, DNA, platelets, VWF, NETs (H3Cit), leukocytes and microcalcifications. H&E staining (**a**) was used to visualize the overall thrombus composition and organization, indicating the presence of an RBC-rich area in red (**b**) and a large DNA-rich area in purple (**c**). Feulgen staining, a specific DNA stain, confirmed the presence of extracellular DNA with few intact nuclei in this area ((**d**), pink). MSB staining and the platelets staining revealed the presence of fibrin ((**e**), red) and platelets ((**f**), purple) on the surface of the thrombus, whereas VWF ((**g**), purple) was present throughout the DNA-rich area. H3Cit staining (purple) showed the presence of extracellular H3Cit positive smears ((**h**), purple)and leukocyte staining showed a large smear with little to no intact leukocytes present ((**i**), purple), indicating that extracellular DNA originated from leukocytes and to some extent from neutrophils. Von Kossa staining showed abundant microcalcifications ((**j**), black). A representative image of anisotype control for the VWF staining is shown in panel (**k**). Scale bars are 500 μm for (**a**) and 125 μm for (**b**–**k**). Reproduced from [27], CC BY 4.0.

A complex internal structure of a blood clot removed from a patient with a right middle cerebral artery M1-occlusion [27] is shown in Figure 2. Areas enriched in red blood cells appeared in specific fragments (Figure 2b). Most of the thrombus consisted of densely DNA-rich material with minimal RBCs (Figure 2c,d). Fibrin was predominantly located at the periphery rather than in the DNA-rich inner core (Figure 2e). Platelets were likewise concentrated along the outer rim (Figure 2f). High levels of von Willebrand factor were present within the central DNA-rich regions (Figure 2g).

Pathological formation of a blood clot within the vascular lumen can be described by Virchow’s triad of the main categories of triggers: endothelial injury, abnormal blood flow, and hypercoagulability. In arterial thrombosis, rupture of an atherosclerotic plaque or endothelial erosion exposes tissue factor and subendothelial collagen, which in turn initiates platelet activation and coagulation cascade. In contrast, venous thrombosis is typically driven by blood stasis and hypercoagulability, but the final outcome in both settings is the formation of an intravascular clot. The composition of the resulting thrombus varies depending on its location and underlying pathology [28,29]. Clots from patients with venous thromboembolism are primarily composed of fibrin with densely packed red blood cells and relatively few platelets [30,31]. In contrast, clots retrieved from patients with myocardial infarction consisted mainly of fibrin and platelets [32]. Thrombi obtained from patients with acute ischemic stroke often exhibit a heterogeneous architecture with alternating regions enriched in either red blood cells or platelets [25].

### 1.2. Biochemical Mechanisms of Thrombolysis Regulation

**Figure 3:**
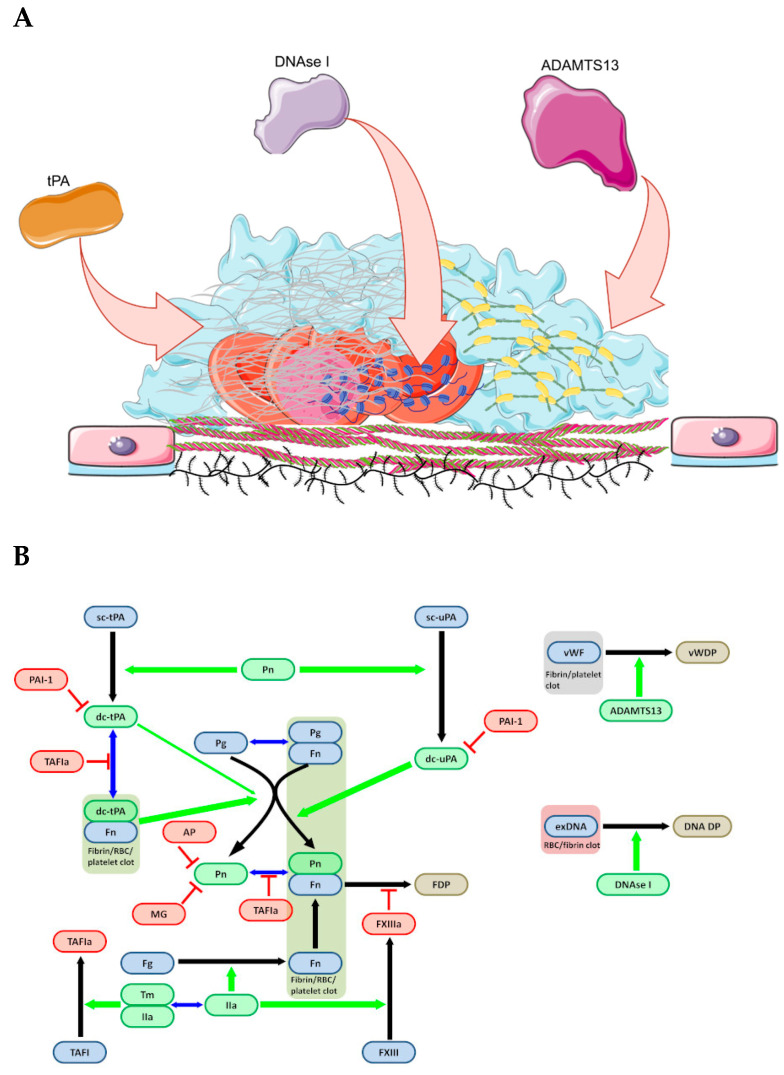
(**A**) Clot structure. New aims for new thrombolytic agents. tPA attacks fibrin rich regions (shown with gray mesh); DNAse I attacks NETs regions rich of extracellular DNA (shown with blue threads); ADAMTS13 attacks vWF rich regions (shown with gray–yellow strands). (**B**) Scheme of thrombolysis reactions. Inactive precursors-zymogens are shown in blue, activated enzymes are shown in green. Inhibitors are shown in red. Black arrows show the transition from the inactive to the active form, blue arrows show the reversible formation of complexes, green arrows indicate that the reaction is catalyzed by this enzyme. Abbreviations: Pg—plasminogen; Pn—plasmin; Fg—fibrinogen; Fn—fibrin; sc-tPA—single-chain form of tissue plasminogen activator; sc-uPA—single-chain form of urokinase plasminogen activator; IIa—thrombin; Tm—thrombomodulin; PAI-1—plasminogen activator inhibitor type 1; AP—antiplasmin; MG—macroglobulin; TAFI(a)—thrombin-activated fibrinolysis inhibitor (active); FDP—fibrin degradation products; vWF—von Willebrand factor; vWDP—degradation products of vWF; exDNA—extracellular DNA in NETs; DNA DP—degradation products of DNA.

Schematic illustrations of clot architecture and the spatial distribution of thrombolytic targets are shown in Figure 3A, while the major biochemical reactions involved in thrombolysis are summarized in Figure 3B. Plasminogen activators convert plasminogen into plasmin. Tissue plasminogen activator (tPA) functions in a fibrin-dependent manner, requiring both tPA and plasminogen to bind to fibrin for efficient activation. In contrast, urokinase (uPA) activates plasminogen independently of fibrin. Plasmin cleaves fibrin within the fibrillar structure of the clot, leading to progressive degradation of the fibrin network. The activities of plasminogen activators and plasmin are tightly regulated by endogenous inhibitors. PAI-1 suppresses tPA and uPA, while antiplasmin and a2-macroglobulin neutralize free plasmin. Fibrin protects fibrin-bound tPA and plasmin from rapid inactivation. But such specific inhibitor as TAFI, activated by thrombin-thrombomodulin complexes, removes C-terminal lysine residues from fibrin. This reduces the binding of plasminogen and tPA to fibrin and thereby attenuates fibrinolysis [33]. As thrombolysis progresses, fibrin becomes increasingly resistant to degradation because cross-linking mediated by factor XIIIa. Factor XIII circulates in plasma as an inactive precursor and is converted to its active form XIIIa by thrombin in the presence of fibrin. Factor XIIIa, a transglutaminase, forms isopeptide bonds between glutamine and lysine residues on adjacent fibrin molecules. These cross-links significantly increase clot density and mechanical stability. Because isopeptide bonds resist cleavage by plasmin and other proteases, degradation of cross-linked fibrin generates D-dimers, in which two fibrin D-domains remain connected via an isopeptide bond. The formation of these cross-links is a significant barrier to the enzymatic dissolution of mature thrombi.

Large vWF multimers can form a fibrin-independent scaffold within thrombi that is not susceptible to plasmin. However, metalloproteinase ADAMTS13 can cleave and dismantle these vWF structures, enabling dissolution of vWF-rich regions of the clot.

Similarly, extracellular DNA within the thrombus, largely originating from NETs, forms structures that are highly resistant to plasmin and ADAMTS13. These DNA strands can be degraded by DNase I, promoting breakdown of regions of the clot that are otherwise refractory to fibrinolysis.

### 1.3. Thrombolysis: Definition and Objectives

Thrombolysis is the pharmacological dissolution of intravascular clots with the goal of restoring blood perfusion. Its primary objective is the enzymatic degradation of fibrin, the key structural component of the clot, thereby destabilizing the clot and allowing blood flow to resume. Successful reperfusion can prevent irreversible tissue injury and reduce both mortality and long-term disability. In ischemic stroke, timely recanalization helps preserve the ischemic penumbra; in myocardial infarction, it maintains myocardial contractile function; and in pulmonary embolism, it reduces right ventricular strain and prevents hemodynamic collapse.

However, recanalization is accompanied by risks, most notably intracerebral and systemic hemorrhage. These complications may arise from systemic depletion of coagulation factors [34], or from hemorrhagic transformation due to blood–brain barrier disruption. The latter is mediated in part by the activation of extracellular matrix metalloproteinases, which compromise vascular bed integrity [35,36].

Thrombolytic therapy is currently used in several acute conditions. In acute ischemic stroke, intravenous recombinant tPA (alteplase) administered within 4.5 h of symptom onset remains the standard of care, with a potent alternative, tenecteplase, a bioengineered variant of alteplase with greater fibrin specificity and a longer half-life [37,38]. In myocardial infarction, fibrinolytic therapy is recommended within 12 h of symptom onset when primary percutaneous coronary intervention cannot be performed within 120 min of diagnosis and no contraindications are present [39]. Thrombolysis is also recommended in high-risk pulmonary embolism [40]; it should preferably be initiated within 48 h of symptom onset but may still provide benefit in selected patients with 6–14 days of symptoms [41]. In venous thrombosis, catheter directed thrombolysis is generally reserved for patients at the highest risk of developing post-thrombotic syndrome, given the substantial risk of bleeding [42].

### 1.4. From Classical to Novel Strategies: Rationale for Innovation

The classical approach of thrombolysis, centered on fibrin degradation by tPA and its derivatives, has demonstrated significant clinical benefit. However, important limitations, including a narrow therapeutic window, incomplete reperfusion, and the risk of major bleeding, underscore the need for innovative strategies. New data on the structure of the clots, derived from patients, shows that clot composition strongly influences responsiveness to fibrinolytic therapy.

Expanding thrombolysis beyond fibrin degradation to include targets such as platelet, vWF, and NETs components, offers new possible pathways for improving outcomes. Additional strategies involve engineered plasminogen activators with enhanced pharmacokinetic properties and resistance to endogenous inhibitors, developing localized delivery systems to minimize systemic toxicity, and designing combination therapies that exploit complementary mechanisms of action.

Taken together, these approaches show the need for next-generation thrombolytic strategies that account for thrombus heterogeneity and the complexity of possible side effects, including mechanisms that extend beyond the classical coagulation and fibrinolytic pathways.

## 2. Classical Approaches to Fibrinolysis: Plasminogen Activators

Fibrinolysis is the enzymatic process by which fibrin clots are broken down to restore vessel patency once they are no longer needed. Although this process is tightly regulated under physiological conditions, pharmacological activation of fibrinolysis has become one of the treatment strategies for several thrombotic diseases, most notably ischemic stroke and acute myocardial infarction. Direct administration of plasmin has been explored but proved clinically impractical due to its short half-life and rapid neutralization by α2-antiplasmin [43,44]. As a result, therapeutic strategies have focused on plasminogen activators, which catalyze the conversion of plasminogen into the active enzyme plasmin.

Over the past half century, three major classes of plasminogen activators have been used: streptokinase (SK), urokinase, and tissue-type plasminogen activator. These agents differ in their mechanism of action, fibrin specificity, clinical efficacy, and adverse effect profiles.

### 2.1. Streptokinase: The First-Generation Plasminogen Activator

Streptokinase, discovered in the 1930s as a fibrinolytic product of Streptococcus cultures, was the first plasminogen activator in clinical use [45]. Unlike proteolytic enzymes, SK is not a serine protease itself. Instead, it forms a 1:1 complex with plasminogen, inducing a conformational change that exposes the active site of plasminogen. This complex then converts additional plasminogen molecules into plasmin by cleaving the Arg561-Val562 bond [46].

Early clinical trials in the 1970s–1980s showed that intravenous SK reduced mortality in acute myocardial infarction when given within 3 h of symptom onset [47]. The GISSI trial [48], which included over than 11,000 patients, demonstrated 8% reduction in 21-day mortality compared with controls. The magnitude of benefit depended on time to treatment, a finding later confirmed in additional trials [49].

Despite its therapeutic effects, SK has important limitations. As a bacterial protein, it is highly immunogenic and frequently elicits allergic reactions or neutralizing antibodies that preclude repeated administration [50]. Moreover, SK lacks fibrin specificity and therefore activates the plasminogen systemically, leading to degradation of circulating fibrinogen, coagulation factors, and extracellular matrix proteins such as collagen. This nonselective activity markedly increases bleeding risk [51,52]. By the late 1990s, SK had been largely replaced by more specific agents in developed countries, although it remains in use in resource-limited settings due to its low cost [53].

### 2.2. Urokinase: A Human-Derived Direct Plasminogen Activator

Urokinase, originally purified from human urine and later produced recombinantly, directly converts plasminogen into plasmin [54]. Physiologically, uPA exists as a single-chain precursor (scuPA, or pro-urokinase) and a two-chain active form (tcuPA). A key component of the urokinase system is the urokinase-type plasminogen activator receptor (uPAR), which localizes plasmin generation to cell surfaces. Binding of scuPA or tcuPA to uPAR concentrates plasmin activity within pericellular regions and promotes focal fibrinolysis; however, this mechanism does not confer fibrin specificity within intravascular thrombi.

Unlike SK, uPA is non-immunogenic and does not induce antibody formation. It was approved for the systemic treatment of pulmonary embolism and for catheter-directed thrombolysis in acute myocardial infarction, with studies demonstrating a significantly lower rate of bleeding complications in patients treated with uPA compared with SK [55].

Nevertheless, uPA, like SK, lacks fibrin specificity and activates the plasminogen systemically, leading to fibrinogenolysis [56], although to a lesser extent than SK [55]. After the introduction of tPA, which showed superior efficacy, the clinical use of uPA declined. However, emerging evidence suggests that uPA may be at least as safe and effective as tPA in acute ischemic stroke [57] and myocardial infarction [58].

### 2.3. Tissue-Type Plasminogen Activator: The Gold Standard

The discovery and cloning of tissue-type plasminogen activator in the 1980s [59] marked a new era in fibrinolytic therapy. Unlike SK and uPA, tPA exhibits high fibrin specificity: it preferentially activates plasminogen when both molecules are bound to fibrin. This confines plasmin generation to the site of the thrombus and reduces systemic fibrinogenolysis. Recombinant tPA became the first biotechnologically engineered thrombolytic drug to gain widespread clinical approval.

In acute myocardial infarction, large-scale trials demonstrated that accelerated tPA infusion reduced 30-day mortality compared with SK [60]. In ischemic stroke, the NINDS trial showed that intravenous tPA administered within 3 h of onset significantly improved 3-month functional outcomes [3], and subsequent trials extended the treatment window to 4.5 h [4]. In pulmonary embolism, tPA therapy was associated with reduced all-cause mortality but an increased risk of major bleeding and intracranial hemorrhage (ICH) [61].

In acute deep-vein thrombosis (DVT), both local and systemic thrombolytic therapy achieved better short- and long-term clinical outcome than conventional anticoagulation therapy alone (with vessel recanalization rates of 37% in control vs. 49% with local thrombolysis and 59% with systemic thrombolysis), though this benefit came at the cost of a substantially increased major bleeding and pulmonary embolic comlications [62].

Hemorrhage remains the major adverse effect of fibrinolytic therapy. In acute myocardial infarction trials, intracranial hemorrhage occurs in 0.5–1% of patients treated with tPA [60]. In ischemic stroke, the rate of symptomatic ICH is 6–7% [3]. These complications may result from systemic fibrinogen depletion, degradation of extracellular matrix proteins, activation of matrix metalloproteinases, and disruption of the blood–brain barrier [36]. Another important adverse effect of tPA is neurotoxicity [4,36,63]. tPA can bind to N-methyl-D-aspartate (NMDA) receptors, increasing calcium influx and promoting excitotoxic neuronal injury [64].

### 2.4. Next-Generation Variants

Because of the limitations in efficacy, safety and practical applicability of the tPA-based thrombolytic therapy, several engineered variants with modified pharmacokinetics and biochemical properties have been developed and clinically evaluated.

Reteplase is a truncated form of tPA with a fourfold longer half-life, a 2–3 fold lower affinity for fibrin, and a fourfold reduction in plasminogenolytic activity [65]. In the RAPID II trial [66], which included 324 patients with acute myocardial infarction, reteplase (administered as a double bolus of 10 + 10 MU) was compared with front-loaded alteplase, with all patients receiving adjunctive heparin and aspirin. Early total patency (TIMI 2–3 flow) was significantly higher in the reteplase group (83.4%) than in the alteplase group (73.3%, *p* = 0.031), whereas late overall patency rates were similar (89.1% vs. 90.3%). These findings indicate that reteplase provides efficacy at least comparable to alteplase, with the additional advantage of simple double-bolus administration.

Tenecteplase is an engineered tPA variant that incorporates point mutations conferring a 14-fold increase in fibrin specificity, an 80-fold increase in resistance to PAI-1, and a 2–4-fold prolongation of half-life [67], enabling single-bolus administration. In acute ischemic stroke, tenecteplase has demonstrated non-inferior or in some settings superior outcomes compared with alteplase. In the NOR-TEST trial [68] (*n* = 261), tenecteplase 0.4 mg/kg produced similar rates of favorable 90-day outcomes (mRS 0–1) across moderate and severe stroke subgroups, although mortality was higher in the tenecteplase arm. More recently, the ORIGINAL trial [37] (*n* = 1489) showed that tenecteplase 0.25 mg/kg was non-inferior to alteplase for excellent functional outcome (72.7% vs. 70.3% achieving mRS 0–1 at 90 days), with comparable safety and slightly lower mortality (4.6% vs. 5.8%). In intermediate-risk pulmonary embolism, however, tenecteplase is not recommended because of an increased risk of bleeding (<30 days: RR = 1.79, 95% CI 1.61–2.00; ≥30 days: RR = 1.28, 95% CI 0.62–2.64) [69]. Overall, available data support tenecteplase as an effective alternative to alteplase, offering comparable efficacy and safety while providing the practical advantage of single-bolus administration.

Desmoteplase, a genetically engineered version of the fibrinolytic protein found in the saliva of the South American vampire bat *Desmodus rotundus* [70], exhibits high fibrin specificity and negligible neurotoxicity in animal models [71]. In patients with acute massive pulmonary thromboembolism, desmoteplase at doses of 180–250 μg/kg demonstrated efficacy comparable to or greater than that of alteplase 100 mg in a small study [72] (*n* = 34). In the DIAS program [73] (*n* = 795), desmoteplase did not provide significant clinical benefit when administered 3–9 h after stroke onset; however, it did not increase the rate of symptomatic intracranial hemorrhage (3.1% vs. 2.6% with placebo), consistent with its low hemorrhagic risk profile and with findings from studies comparing it indirectly to alteplase. Although the overall clinical results have been mixed, desmoteplase has demonstrated a consistently favorable safety profile.

Several additional modified tPA variants with increased half-life, including monteplase [74], lanoteplase [75], and pamiteplase [76], have shown efficacy comparable to or slightly greater than that of tPA, with similar or more favorable bleeding profiles.

## 3. Strategies to Improve Thrombolytic Therapy: Expanding Indications and Reducing Side Effects

The limitations of classical fibrinolytic therapy, including narrow therapeutic windows, incomplete recanalization of resistant thrombi, and significant hemorrhagic risk, have driven the development of next-generation strategies aimed at enhancing efficacy, broadening applicability, and improving safety. These approaches fall into several overlapping categories: targeting non-fibrin structural components of thrombi, using combinatorial and adjunctive agents, implementing advanced drug-delivery systems, engineering novel or modified plasminogen activators, blocking endogenous fibrinolysis inhibitors, exploring alternative proteolytic enzymes, and personalizing therapy based on clot composition. The main strategies, currently used in clinical practice or under research are briefly summarized in Table 1.

### 3.1. Targeting Neutrophil Extracellular Traps to Improve Thrombolysis

DNAse I, an endonuclease that hydrolyzes extracellular DNA, has been extensively investigated as a potential adjunctive therapy aimed at degrading the DNA backbone of NETs, thereby enhancing fibrinolytic access. Experimental models consistently demonstrate that DNase I promotes thrombus disintegration, enhances tPA penetration, and accelerates overall clot lysis kinetics [77,78]. In a mouse photothrombotic model of ischemic stroke, administration of DNase I, but neither tPA nor the combination of tPA and DNAse I significantly reduced infarct size [79]. DNAse I alone has also been shown to be safe and effective treatment in experimental ischemic stroke in mice [80].

The combination of DNAse I and tenecteplase significantly restored cerebral blood flow, reduced infarct volume, and resulted in less severe neurological deficits compared with tenecteplase alone [81].

Beyond direct DNA degradation, DNAse I may exert additional benefits by reducing the release cytotoxic NET-associated components such as histones [82]. Histones can induce endothelial injury and platelet activation [83], and their removal through NET disruption may therefor provide dual advantages: facilitating clot dissolution while protecting vascular endothelium integrity [84].

Although DNAse I has long been approved for clinical use in cystic fibrosis (as recombinant human DNAse I, dornase alfa [85]), its translation to thrombolytic therapy remains at an early stage. Pilot clinical studies are ongoing to evaluate its safety and efficacy in thrombotic conditions. A phase 2 trial “Improving Early Reperfusion with Adjuvant Dornase Alfa in Large Vessel Ischemic Stroke” (NCT05203224) is currently in progress. In this study, patients with acute ischemic stroke due to large-vessel occlusion (ICA, M1, M2, or basilar artery) receive a single intravenous dose of dornase alfa (0.125 mg/kg, 0.25 mg/kg, 0.5 mg/kg or 1 mg/kg in escalating tiers), administered as a 30 s bolus. The primary endpoint is the proportion of patients achieving substantial angiographic reperfusion or having no retrievable intracranial thrombus at initial angiogram without symptomatic intracerebral hemorrhage.

Combination therapy of DNAse I and plasminogen activators may also enable dose reduction of fibrinolytic agents, potentially lowering the risk of hemorrhagic complications, one of the major limitations of conventional uPA/tPA-based therapy.

### 3.2. Targeting Von Willebrand Factor to Improve Thrombolysis

Histopathological analyses of thrombi retrieved from ischemic stroke patients indicate that vWF is a common and quantitatively significant component of cerebral thrombi [25]. In a cohort of 36 human thrombi, vWF was detected in all samples and accounted for approximately 20% of thrombus area. Its abundance was inversely correlated with red blood cell content and positively correlated with fibrin content [86]. In the same study, tPA was ineffective against vWF-rich thrombi in a mouse stroke model, whereas administration of ADAMTS13 successfully dissolved these tPA-resistant clots and reduced infarct volumes. These findings demonstrate that vWF contributes directly to the clot resistance to tPA in vivo. Additional clinical evidence supports the role of vWF in thrombus stability and clinical severity. In a study of 131 patients with acute ischemic stroke undergoing thrombectomy, both plasma vWF levels and the vWF:ADAMTS13 ratio (often reflecting endothelial activation and proteolytic imbalance) correlated with poorer clinical outcomes [87]. Patients with higher NIH Stroke Scale scores had elevated vWF and higher vWF:ADAMTS13 ratios, and immunohistochemical analysis of retrieved thrombi showed that increased vWF content was associated with greater platelet and fibrin accumulation.

In experimental models, cleavage of vWF by ADAMTS13 has been shown to reduce infarct size and improve functional outcomes. In vWF-deficient mice, infarct volumes after cerebral ischemia were significantly smaller than in wild-type animals [88,89], and administration of recombinant human ADAMTS13 reduced infarct volume, improved motor outcomes, and did not induce hemorrhage, unlike tPA [88]. Recombinant ADAMTS13 also attenuated tPA-induced blood–brain barrier disruption and hemorrhage in murine stroke, likely through cleavage of vWF and subsequent reduction of vascular endothelial growth factor upregulation and MMP-9 activation triggered by tPA [90]. Together, these findings highlight both the thrombolytic and protective effects of targeting vWF in ischemic injury.

A constitutively active variant of ADAMTS13 (caADAMTS13), carrying an Ala1144Val substitution, demonstrated particularly potent activity. At subphysiological concentrations (<5 nM), caADAMTS13 completely inhibited vWF-mediated platelet adhesion under arterial shear conditions, enhanced tPA-induced fibrinolysis, and effectively restored cerebral blood flow following distal MCA occlusion [91]. Subsequent studies suggest even greater efficacy, indicating that caADAMTS13 may provide adjunctive thrombolysis in arterial occlusions that are otherwise refractory to tPA [92].

Modulating vWF levels or activity, by reducing ultralarge multimer formation, blocking platelet-vWF interactions, or limiting endothelial vWF release, may synergize with tPA. In stroke models, pharmacologic blockade of vWF-platelet binding using an anti-vWF aptamer, which selectively inhibits vWF-mediated platelet adhesion, promoted vascular recanalization of platelet-rich thrombotic occlusions in murine and canine carotid arteries [93]. Agents such as N-acetylcysteine (NAC), which reduces disulfide bonds within vWF multimers, have also been shown to facilitate lysis of platelet-rich thrombi in large-vessel thromboembolic stroke model in mice [94]. The safety of NAC as an adjunct to tPA therapy was evaluated in a small, single-group clinical study [95]. Although NAC was generally well tolerated (no severe adverse events in 80% of the participants), two fatal intracranial hemorrhages occurred in patients with prior antiplatelet therapy, underscoring the need for caution and further evaluation.

Another strategy is the use of thrombolytic agents that require vWF binding for plasminogen activation. One such example is Microlyse, a single polypeptide consisting of a vWF-targeting nanobody and the catalytic domain of urokinase plasminogen activator [96]. In this work, a mouse model of thrombotic thrombocytopenic purpura was used to demonstrate that Microlyse removed microthrombi significantly faster than caplacizumab and ameliorated thrombocytopenia and tissue injury in ADAMTS13^−^/^−^ mice without increasing bleeding. In murine ischemic stroke models where fibrin-rich thrombi were induced by thrombin infusion, and platelet-rich thrombi were generated by topical FeCl3 application Microlyse was noninferior to tPA in fibrin-rich occlusions but demonstrated superior efficacy in platelet-rich stroke models [97].

### 3.3. Targeting Extracellular Matrix Components and Proteoglycans to Enhance Thrombolysis

Beyond the canonical fibrin-platelet framework, thrombi contain a complex extracellular matrix (ECM) whose composition that strongly influences their mechanical stability, porosity, and susceptibility to fibrinolysis. Although fibrin and platelet aggregates constitute the core structural elements of a clot, the surrounding ECM, comprising proteoglycans, glycosaminoglycans (GAGs), fibronectin, laminin, and collagen fragments, acts as an additional biochemical and biomechanical scaffold that modulates clot remodeling and limits access of fibrinolytic agents. Understanding and targeting ECM components may therefore improve the dissolution of thrombi that are resistant to tPA.

GAGs and proteoglycans exert diverse and sometimes opposing effects on fibrinolysis. Heparan sulfate proteoglycans bind antithrombin [98], reducing local thrombin activity and thereby indirectly affecting fibrin cross-linking density. ECM *g*lycoproteins such as fibronectin and vitronectin provide adhesion sites that stabilize active PAI-1 on subendothelium [99], locally downregulating fibrinolysis and tPA-mediated pathways of neuroinflammation. These proteins also promote platelet adhesion to the subendothelium and enhance thrombogenesis on non-fibrillar type I collagen surfaces [100]. Heparan sulfate and chondroitin sulfate proteoglycans can act as reservoirs for tPA and plasminogen [101,102], promoting plasmin activation [103], while at the same time sequestering it away from fibrin surfaces. ECM proteins such as fibronectin and laminin may also cross-link with fibrin via factor XIIIa, generating hybrid fibers that are more resistant to plasmin-mediated degradation [104]. In contrast, dermatan sulfate exerts pro-fibrinolytic effects: it induces tPA release from endothelial cells in vivo [105], decreases PAI-1 level in cultured human umbilical vein endothelial cells [106], and enhances tPA-mediated plasminogen activation [107], thereby facilitating fibrinolysis.

Proteoglycans can serve as substrates for matrix metalloproteinases and neutrophil elastase, releasing degradation fragments that promote further platelet activation and local inflammation, thereby reinforcing the thrombus rather than weakening it [108].

Thrombus composition also changes over time. In a mouse model of venous thrombus remodeling, fibrin was gradually replaced by collagen between 2 and 4 weeks after thrombus formation, accompanied by infiltration of inflammatory and mesenchymal cells into the clot [109].

Several animal-derived venoms with hemorrhagic activity exhibit both fibrinolytic properties and the ability to degrade extracellular matrix components. Enzymes from *Bothrops jararaca*, *Bothrops atrox* venoms, lonomin V, derived from hemolymph of *Lonomia achelous* caterpillar cleave laminin, entactin, type IV collagen, fibronectin, vitronectin and fibrin [110,111]. In a rabbit jugular vein thrombosis model lonomin V demonstrated thrombolytic efficacy comparable to tPA [112], and Batroxase (a metalloprotease from *Bothrops atrox* venom) showed similar efficacy to tPA in a rat venous thrombosis model [113]. At least part of their thrombolytic effect is likely attributable to degradation of ECM proteins.

However, systemic degradation of ECM carries significant risks, including vascular fragility, blood–brain barrier disruption, and hemorrhage. Therefore, the potential use of ECM-degrading agents may only become clinically viable when combined with site-specific and temporally controlled delivery systems, such as ultrasound-activated carriers or catheters releasing localized doses drugs.

### 3.4. Destabilase: Targeting Fibrin Isopeptide Cross-Links to Facilitate Thrombolysis

One of the mechanisms to improve thrombolysis is to target the isopeptide cross-links that stabilize mature, “aged” fibrin clots. These cross-links, formed by Factor XIIIa, make fibrin resistant to plasmin-mediated degradation and are a key reason older thrombi are poorly lysed by classical plasminogen activators.

An enzyme with the ability to cleave the isopeptide bonds in the fibrin clot is destabilase from the leech Hirudo medicinalis. In the first half of the 1980s, it was discovered that the secretion of the leech salivary glands (SGS) has thrombolytic activity but lacks proteolytic activity. Furthermore, it was noted that the higher the stabilization of the thrombus, the more pronounced the thrombolytic properties of the SGS [114]. In 1985, the enzyme destabilase was isolated, and it was shown that it hydrolyzes isopeptide bonds in thrombi [115]. Subsequent research determined the sequence of cDNAs encoding destabilase and produced the recombinant protein [116,117]. Analysis of the primary structure of destabilase showed that it belongs to lysozymes; this enzyme became the first described lysozyme of the new i-type [118]. Later, three recombinant isoforms of destabilase were expressed in E. coli, and their enzymatic properties were characterized in detail [119]. All three isoforms exhibited similar properties, demonstrating both isopeptidase and muramidase activities, as well as non-enzymatic antibacterial effects.

Despite the presence of all expected activities in recombinant destabilase, questions remained regarding how closely these proteins resembled the native enzyme. The problem is that the structure of destabilase contains seven conserved cysteine residues, and in E. coli, this protein accumulates in inclusion bodies in an inactive form and requires refolding. In this regard, recombinant destabilase was also produced in other expression systems—human Expi293 cell culture and the yeast Pichia pastoris [120]. As a result, destabilase is produced in a soluble active form. Both the muramidase and isopeptidase specific activities of the soluble destabilase in this case were an order of magnitude higher than the activities of the protein obtained in E. coli. Circular dichroism spectroscopy showed that refolded destabilase has, on average, a disordered structure, while the soluble form has a predominantly alpha-helical structure. Thus, in the composition of refolded destabilase, only a few percent of all molecules have the correct fold. Therefore, despite economic considerations, for potential clinical applications, destabilase should be produced in human cell culture or yeast.

**Figure 4:**
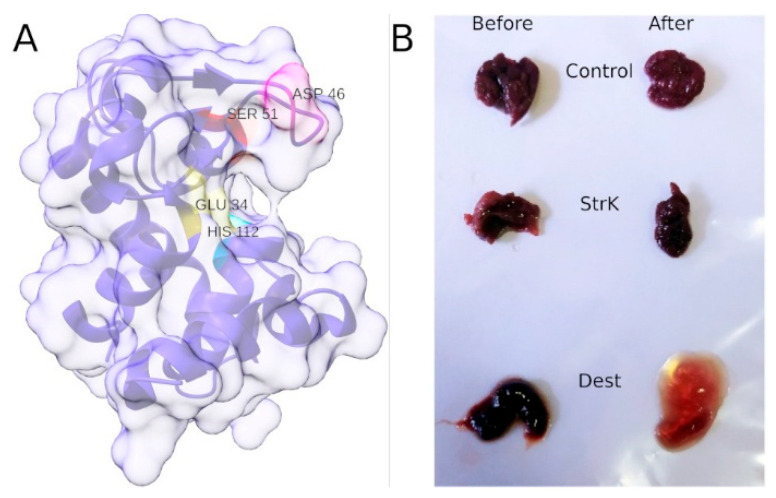
(**A**) Structure of destabilase with labeled catalytic amino acid residues. The muramidase catalytic dyad consists of residues Asp46 (magenta) and Glu34 (yellow). The putative isopeptidase catalytic triad [121] includes residues Ser31 (red), His112 (cyan), and the same Glu34 residue that is part of the muramidase dyad. All residues are grouped around a deep cavity on the molecular surface. Asp46 is located in a flexible loop capable of changing its position within wide limits. The image was generated based on the structure PDB ID: 8bbw using UCSF ChimeraX 1.10.1. (**B**) Blood clot morphology change after destabilase treatment. The dry blood clot was divided into parts, incubated with destabilase (Dest), streptokinase (StrK), and control buffer (Control) for 24 h, and lyophilized. Then, three parts were incubated in 2% acetic acid solution for the next 24 h, and an additional three parts were rehydrated in 2% acetic acid for 10 min prior to photographing. Panel (**B**) reproduced from [122], CC BY 4.0.

Obtaining correctly folded destabilase made it possible to determine its spatial structure using X-ray structural analysis [121]. Destabilase, as expected, is a predominantly alpha-helical protein with a lysozyme-like fold. The muramidase and putative isopeptidase active sites are located within a single cavity on the molecular surface and partially overlap (Figure 4A).

However, the presence of isopeptidase activity in vitro does not necessarily confirm that destabilase can cleave isopeptide bonds in actual thrombi. In this regard, studies were conducted on its effect on real human thrombi extracted during surgery [122]. Each thrombus was divided into three parts and treated with destabilase, streptokinase, or buffer alone. The results demonstrated that fresh and aged thrombi responded differently (Figure 4B). Thrombus fragments that lost more mass upon treatment with destabilase lost less when treated with streptokinase, and vice versa. Older thrombi were more effectively degraded by destabilase, whereas atherosclerotic plaque exhibited high resistance in all cases.

To demonstrate the thrombolytic effect of destabilase in vivo, a model of induced arterial and venous thrombosis in rats was used [123]. The study showed that destabilase caused a 47.6% and 74.6% reduction in the mass of venous and arterial thrombi, respectively. The enzyme proved more efficient in dissolving thrombi than streptokinase. Moreover, combined administration of destabilase and streptokinase produced a synergistic effect, exceeding that of either enzyme alone. Destabilase reduces fibrin stabilization within thrombi.

Destabilase was the first enzyme found with isopeptidase activity. Subsequently, isopeptidase activity was also discovered in other invertebrate lysozymes [121]. However, among these invertebrates, only leeches are blood-feeding organisms that come into contact with mammalian blood. Recently, destabilase has also been described in other leech species besides H. medicinalis. However, they are either described purely bioinformatically [124], or at the level of primary in vitro experiments to demonstrate the presence of enzymatic activity [125]. Thus, this enzyme from H. medicinalis remains the most studied representative of destabilases.

Because destabilase action is not proteolytic in the broad sense, it may minimize systemic bleeding risks while extending the effective therapeutic window beyond that of classical PAs.

### 3.5. Inhibition of Fibrinolysis Inhibitors as a Strategy to Enhance Thrombolytic Efficacy

Pharmacological inhibition of PAI-1 has been explored as a strategy to enhance the efficacy of thrombolysis. A range of small-molecule inhibitors, peptides, and monoclonal antibodies has been developed to block PAI-1 activity through various mechanisms, including stabilization of its latent conformation, prevention of PAI-1/tPA complex formation, or acceleration of its spontaneous inactivation [126]. Among small-molecule compounds, tiplaxtinin [127] was one of the first orally bioavailable inhibitors shown to reduce thrombus weight in vivo in a rat model of thrombosis [128] and to induce spontaneous reperfusions in a canine model of coronary artery thrombosis, as well as accelerate clot lysis ex vivo [129]. Similar effects have been reported for TM5275, which demonstrated antithrombotic activity in a rat FeCl3-induced carotid artery thrombosis model. When combined with a low dose of tPA (0.3 mg/kg), TM5275 significantly enhanced the antithrombotic effect of tPA and produced a benefit comparable to that of a high tPA dose (3 mg/kg), without increasing bleeding time [130].

Neutralizing monoclonal antibodies such as MA33H1 in a dose of 6 mg/kg reduced cerebral infarct volume by 50% in a mouse model of transient middle cerebral artery occlusion [131], likely through enhancement of endogenous fibrinolysis.

Elevated plasma levels or enhanced activation of TAFI have been associated with hypofibrinolytic states and an increased risk of thrombotic events such as deep-vein thrombosis and stroke [132,133]. Conversely, TAFI deficiency or pharmacological inhibition produces a pro-fibrinolytic phenotype characterized by accelerated clot lysis and reduced thrombus persistence [134]. These observations suggest that targeting TAFI may enhance endogenous fibrinolysis without markedly disrupting hemostatic balance.

Nanobodies that inhibit TAFIa activity or block thrombin/thrombomodulin-mediated or plasmin-mediated activation of TAFI, have demonstrated profibrinolytic properties in clot lysis in vitro experiments [135]. Owing to their smaller size, these nanobodies penetrate clot more efficiently than conventional inhibitory monoclonal antibodies.

Neutralizing antibodies such as MA-TCK26D6, directed against the active form of TAFI, significantly enhanced fibrinolysis in a mouse thromboembolism model [136] and improved cerebral blood flow and reduced cerebral fibrin(ogen) deposition and infarct sizes by 50% in a mouse model of transient middle cerebral artery occlusion [131].

### 3.6. Reduction of Plasminogen Activator Therapy Side Effects

Efforts to mitigate the bleeding complications of thrombolytic therapy have focused on increasing fibrin specificity and reducing systemic plasmin generation, as achieved with third-generation agents such as tenecteplase [67] and desmoteplase [70]. The latter PA variant shows negligible neurotoxicity, likely due to the absence of the K2 domain and the plasmin cleavage site within its protease domain, both of which are required for tPA interaction with NMDA [137].

Neurotoxic effects of tPA can also be neutralized pharmacologically. The PAI-1-derived hexapeptide EEIIMD abolished the tPA-induced increases in infarct size and intracranial bleeding in both mechanical and embolic models of stroke in rats [138,139].

A direct way to improve thrombolysis efficacy while minimizing systemic exposure is localized administration of fibrinolytic agents via catheter or microcatheter. Intra-arterial thrombolysis (IAT) enables delivery of tPA directly into the thrombus under angiographic guidance, achieving higher local concentrations with reduced systemic effects [140]. IAT has been shown to improve recanalization rates in acute ischemic stroke [141,142] and myocardial infarction, particularly when used in combination with mechanical thrombectomy [143,144].

Ultrasound-assisted catheter-directed thrombolysis (USAT), such as the EKOS system, offers an additional method to enhance fibrinolysis by using acoustic cavitation and microstreaming to increase drug penetration into the clot [145]. Clinical trials, including SEATTLE II and OPTALYSE PE, demonstrated that USAT can achieve substantial clot reduction in pulmonary embolism using substantially reduced tPA doses, thereby minimizing bleeding complications [146,147,148].

Nanoparticles enable precise and sustained delivery of thrombolytic agents owing to their tunable surface chemistry, size-dependent biodistribution, and capacity for targeted ligand conjugation. A nanocomposite prepared by heparin-mediated cross-linking of urokinase with magnetite nanoparticles demonstrated efficient lysis of experimental clots both in vitro and in animal models of FeCl3 induced thrombosis, resulting in complete restoration of blood flow [149]. Similarly, magnetic polymeric nanoparticles carrying tPA achieved superior thrombolytic efficacy using only 10–20% of the standard systemic tPA dose [150].

Stimuli-responsive carriers represent an innovative class of delivery systems designed to release thrombolytic agents only in response to specific external triggers. A bioengineered system consisting of cells encapsulated within an injectable hydrogel and equipped with an artificial signaling circuit enabled remote and precise control of uPA production in response to near-infrared light-induced photothermal stimulation. This system demonstrated thrombolytic efficacy in mouse tail thrombosis model [151].

### 3.7. Personalizing Thrombolysis Based on Clot Composition, Origin, and Age

The heterogeneity of thrombi with respect to composition, origin, and degree of maturation is a critical determinant of both mechanical and pharmacological reperfusion success. Personalization of thrombolytic therapy, including choice of agen, dosage, delivery route, and use of adjunctive treatment, based on the specific structural and biochemical characteristics of an individual thrombus may substantially increase the likelihood of a successful clinical outcome.

Histopathological analyses of retrieved thrombi have revealed pronounced variability in the relative proportions of RBCs, fibrin, platelets, leukocytes, NETs, and vWF [25,152]. These compositional differences closely correlate with thrombus etiology and therapeutic responsiveness. For instance, thrombi of cardioembolic origin, commonly formed in the atria during atrial fibrillation, are typically fibrin- and platelet-rich, whereas large-artery atherosclerotic thrombi often contain a higher proportion of RBCs and exhibit a looser structural organization [153,154].

Such variations have important clinical implications. RBC-rich “red” thrombi are generally more porous, permitting greater penetration by tPA and faster lysis, whereas fibrin- and platelet-rich “white” thrombi form denser networks that limit enzymatic access and slow plasmin generation [25].

With aging, thrombi undergoes remodeling processes which include contraction, fibrin cross-linking, and cellular infiltration by leukocytes and fibroblasts [155]. Aged thrombi exhibit markedly reduced responsiveness to fibrinolysis [156]. Experimental studies demonstrate that progressive thrombus compaction over time decreases permeability to tPA and can double clot lysis time, underscoring the importance of considering thrombus age when selecting thrombolytic therapy [157].

**Table 1:** Current pharmacological approaches in thrombolysis.

Therapy	Target	Disease	Side Effects	Advantage	Status	Refs
Streptokinase (SK)	Fibrin	AMI	Immunogenicity systemic activation of plasminogen bleeding	Low cost	Not used in developed countries but used in developing countries	[47,48,49]
uPA	Fibrin	PE AMI AIS	depletion of fibrinogen bleeding	No immunogenicity Lower bleeding	Limited use in AIS	[55,57,158]
tPA	Fibrin	AMI AIS PE	ICH bleeding neurotoxicity	Higher efficacy Lower systemic action	Worldwide standard	[3,60,61]
Tenecteplase (TNK)	Fibrin	AIS AMI	Bleeding	Lower bleeding Better administration profile	Ongoing clinical trials limited use	[37,159]
Desmoteplase	Fibrin	AIS	Bleeding	No neurotoxicity Lower bleeding	Completed clinical trials	[160]
DNAse I DNAse I + tPA DNAse I + TNK	NETs NETs + fibrin NETs + fibrin	AIS	No significant	Better neurological outcome, lower bleeding	In vitro and in vivo studies Clinical trial	[79,80] NCT05203224
Adamts13	vWF	AIS	No significant	Better neurological outcome Lower bleeding	In vivo studies	[88]
caADAMTS13 + tPA	vWF + fibrin	AIS	No significant	Enhanced clot lysis	In vivo studies	[91]
NAC + tPA	Multimer vWF formation + fibrin	AIS	Bleeding in patients with antiplatelet therapy	Enhanced clot lysis	Small group studies	[95]
Destabilase + SK	Fibrin isopeptids cross-links + fibrin	Arterial and venous thrombosis		Enhanced clot lysis	In vitro Animal models	[122,123]
Tiplaxtinin, TM5275 + tPA	PAI-1, fibrin	Arterial thrombosis		Enhanced clot lysis, similar to increased tPA dose	Animal models	[129,130]
MA33H1	PAI-1	AIS		Enhanced endogeneous fibrinolysis, reduced ischemia volume	Animal models	[131]
MA-TCK26D6	TAFI	AIS		Enhanced endogeneous fibrinolysis, reduced ischemia volume	Animal models	[131,136]

AMI—acute myocardial infarction, PE—pulmonary embolism, AIS—acute ischemic stroke, and ICH—intracranial hemorrhage.

## 4. Conclusions

The evolution of thrombolytic therapy reflects a gradual expansion of potential therapeutic targets and areas of applicability, shifting the focus from direct fibrin dissolution towards multiple structural and cellular components of the blood clot. This paradigm shift recognizes thrombosis not merely as a simple local occlusion but as a complex, multicomponent process. It is now evident that the efficacy and safety of thrombolysis depend not only on the activity of fibrinolytic agents but also on their interactions with the broader biological environment of the thrombus and the vessel wall.

The identification of non-fibrin clot constituents, including NETs, vWF, and cellular or extracellular matrix components, has enabled the development of novel therapeutic strategies. These include enzymatic approaches targeting specific clot components, such as DNase I for NET degradation and ADAMTS13 for vWF-mediated platelet aggregates. Identification of coagulation/fibrinolysis imbalance driven by elevated levels or activity of endogenous fibrinolysis inhibitors has motivated using PAI-1 or TAFIs to enhance fibrinolytic efficiency. Modifying clot architecture with agents such as destabilase represents a potential experimental strategy that remains to be validated. While such approaches may theoretically permit the use of lower doses of plasminogen activators or enhance the performance of non-neurotoxic agents such as uPA or desmoteplase, their therapeutic benefit has not yet been demonstrated in preclinical or clinical studies. Rather than replacing established therapies, these approaches are most likely to complement plasminogen activator-based thrombolysis, with the aim of achieving comparable or improved efficacy at lower systemic doses and with a reduced risk of bleeding and neurotoxicity.

Recent advances in biomaterials, nanomedicine, and targeted delivery systems provide opportunities to localize thrombolytic activity more precisely to sites of occlusion, thereby minimizing systemic exposure. In parallel, systems biology and computational modeling are improving our understanding of regulatory pathways, enabling optimization of drug properties, and guiding the design of rational therapeutic combinations.

Future challenges extend beyond the discovery of new agents to their integration into adaptive, patient-specific treatment strategies. Clot composition, age, and etiology vary markedly among individuals and strongly influence therapeutic response. The integration of molecular profiling, high-resolution imaging, and predictive modeling may enable real-time assessment of clot characteristics and support more individualized therapeutic decisions.

In summary, the development of next-generation thrombolytic therapies will rely on coupling mechanistic insight with advances in targeted delivery and quantitative modeling. Such integrated approaches have the potential to improve both the efficacy and safety of thrombolysis and to translate biological understanding into clinically meaningful benefit.

## Data Availability

No new data were created or analyzed in this study. Data sharing is not applicable to this article.

## Abbreviations

The following abbreviations are used in this manuscript:

DNA	deoxyribonucleic acid
ADAMTS13	a disintegrin and metalloproteinase with a thrombospondin type 1 motif, member 13
NETs	neutrophil extracellular traps
DNAse	deoxyribonuclease
vWF	von Willebrand factor
PAI-1	plasminogen activator inhibitor-1
tPA	tissue-type plasminogen activator
uPA	urokinase plasminogen activator
SK	streptokinase
TAFI	thrombin-activatable fibrinolysis inhibitor
RBC	red blood cell
AMI	acute myocardial infarction
PE	pulmonary embolism
AIS	acute ischemic stroke
DVT	deep-vein thrombosis
ICH	intracranial hemorrhage
